# Travel decision making during and after the COVID-2019 pandemic: Revisiting travel constraints, gender role, and behavioral intentions

**DOI:** 10.3389/fpsyg.2022.961464

**Published:** 2022-09-27

**Authors:** Norzalita Abd Aziz, Fei Long, Miraj Ahmed Bhuiyan, Muhammad Khalilur Rahman

**Affiliations:** ^1^UKM-Graduate School of Business, National University of Malaysia, Bangi, Selangor, Malaysia; ^2^Business School, Guangdong Ocean University, Yangjiang, China; ^3^School of Economics, Guangdong University of Finance and Economics, Guangzhou, China; ^4^Faculty of Entrepreneurship and Business, Angkasa-UMK Research Academy, Universiti Malaysia Kelantan, Kota Bharu, Kelantan, Malaysia

**Keywords:** travel constraints, gender, travel intentions, COVID-19, Malaysia

## Abstract

The COVID-19 pandemic has deeply influenced the tourism and hospitality industry, and it has also reshaped people’s travel preferences and related behaviors. As a result, how prospective travelers perceive travel constraints and their effects on future travel behaviors may have changed to some extent. Besides, such perception arguably varies across gender. Therefore, this research examines the interplay between travel constraints, gender, and travel intentions for facilitating robust tourism recovery by revisiting the Leisure Constraints Model (LCM) from a gender perspective. Data were collected through a survey from 357 Malaysian prospective travelers. By conducting path analysis and multigroup analysis (MGA), it is found that structural and interpersonal constraints impose indirect effects on travel intentions (mediated by intrapersonal constraints), and gender moderating the effect of structural cost on intrapersonal constraints and effect of intrapersonal constraints on travel intentions. Based on these findings, this research provides theoretical and practical implications into how to adjust their marketing strategies and travel products during the era of “new normal” for tourism policy makers, destination marketers, and related businesses.

## Introduction

The tourism industry has been severely disrupted by the novel coronavirus 2019 (COVID-19) pandemic in an unprecedented way. Many countries implemented travel restrictions or even border shutdowns to contain the spread of the airborne virus. At one point, travel activities almost ceased to exist in some destinations (WTTC, 2020). Even if many destinations eased travel restrictions, the outlook of the industry remains uncertain. According to McKinsey’s forecast, tourism may not fully recover until 2023, and the total tourism-related GDP losses may be as high as $3–8 trillion in the world (Binggeli et al., 2020). What are worse, prospective travelers may become reluctant to travel out of a variety of concerns even after the end of the current pandemic (Shin et al., 2022). Amid the era of “new normal,” post-crisis management focusing on tourist flow has been intensively discussed by researchers and practitioners for tourism survival and recovery purposes (Rasoolimanesh et al., 2021).

Tourist flow is a complex dynamic system that is collectively shaped by factors related to destinations and travelers (Jin et al., 2019). During the current pandemic, it is imperative for tourism marketers to gain a thorough understanding of all these factors so as to devise effective marketing strategies for restoring tourist flow. However, most relevant literature are mainly written from the angle of destinations (Hassan and Soliman, 2021), and factors related to prospective travelers are insufficiently discussed (Jin et al., 2019; Shin et al., 2022). Meanwhile, the pandemic has arguably made noticeable impacts on people’s travel behaviors, and thus some earlier assumptions on travel decision making are seriously challenged (Kock et al., 2020; Neuburger and Egger, 2020; Wen et al., 2020). Therefore, certain tourism knowledge drawn from previous studies should be further examined in the pandemic context.

Undoubtedly, individuals may perceive undesirable internal obstacles and external barriers restraining their intentions to travel (Crawford and Godbey, 1987). After the outbreak of the COVID-19 in December 2019, travel became more difficult and expensive due to various control measures (e.g., mandatory quarantine). As a result, travel constraints became more salient for prospective travelers (Aziz and Long, 2022). Following the taxonomy proposed by Crawford and Godbey (1987), this research focuses on three major travel constraints, including intrapersonal constraints, interpersonal constraints, and structural constraints. Although there are many prior studies discussing travel constraints, little is known about the interplay of the three major travel constraints and how travel constraints influence travel decision making in the midst of a world health crisis (Pan et al., 2021).

Normally, individuals are less likely to travel if they perceive a high level of travel constraints, but this association may vary depending on gender (Yamashita and Hallmann, 2021). In tourism literature, gender differences concerning travel behaviors are regrettably overlooked (Huang and van der Veen, 2019). However, we do find some evidence suggesting that gender could somehow explain differences of travel behaviors through a comprehensive literature review, including tourists’ perception of travel constraints (Hudson, 2000; Losada et al., 2016; Tavakoli and Mura, 2021). Specifically, a few studies indicate that female travelers are inclined to perceive more risks and constraints when they participate in travel activities due to gender roles expectations (Jackson and Henderson, 1995; Nyaupane and Andereck, 2008; De Pascale et al., 2022). Therefore, it is worthwhile to examine the possible moderating role of gender with regard to the relationship between travel constraints and travel decision making (Khan et al., 2019a).

Given the knowledge gap aforementioned, this research intends to examine the interplay of three major travel constraints and travel intentions, and whether the interplay is moderated by gender in the current crisis context. Specifically, this study (1) investigates how the three major travel constraints affect travel intentions, (2) whether the intrapersonal constraints mediate the relationship between interpersonal/structural constraints and travel intentions, and (3) whether gender moderate the interplay among the three major travel constraints and travel intentions. The findings of the research will provide tourism stakeholders with meaningful insights for tourism survival and recovery.

## Literature review

### Travel constraints and travel intentions

Travel constraints have been widely discussed in tourism literature since Crawford and Godbey (1987) introduced the hierarchical Leisure Constraints Model (LCM). The model is further enhanced by some following research, such as Crawford et al. (1991), Jackson and Dunn (1991), and Jackson (1993). In the past three decades, many studies have adopted the LCM to examine how travel constraints influence travel intentions, and much knowledge has been drawn from these research (Mei and Lantai, 2018; Yang and Tung, 2018; Jian et al., 2021). It is generally recognized that travel constraints, as obstacles and inhibitors, significantly affect individuals’ preferences and participation in tourism activities (Wong and Kuo, 2021). Therefore, most relevant studies assume that travel constraints have a negative impact on travel intentions (Nyaupane and Andereck, 2008; Hung and Petrick, 2012). Significantly, Shin et al. (2022) explicitly state that it is crucial to understand the impacts of travel constraints on travel intentions so as to devise effective marketing strategies for tourism recovery during the COVID-19 pandemic.

Based on the LCM, leisure constraints are made of intrapersonal, interpersonal, and structural constraints (Godbey et al., 2010). Intrapersonal constraints are defined as perceived internal psychological or cognitive barriers, such as stress and anxiety that influence individuals’ behaviors (Crawford and Godbey, 1987). Koronios et al. (2020) concluded that intrapersonal constraints significantly influence consumption behaviors. Similarly, this type of constraints plays a powerful role in determining travel decision making (Karl et al., 2020; Chen et al., 2021). However, Hawkins et al. (1999) argue that intrapersonal constraints may not be a major factor causing non-participation in leisure activities, and Yang et al. (2022) also found that intrapersonal constraints do not directly influence travel intentions. During the COVID-19 pandemic, many prospective travelers tend to worry about their own health and safety with regard to Coronavirus infection (Neuburger and Egger, 2020), and they probably will experience more intrapersonal constraints (Shin et al., 2022). As a result, their travel intentions may decrease during and after the global pandemic. Therefore, the following hypothesis is proposed:

*H1*: Intrapersonal constraints negatively influence travel intentions among Malaysian travelers during the COVID-19 pandemic.

Besides, interpersonal constraints could also have a negative impact on prospective travelers’ intentions to travel. According to Crawford and Godbey (1987), interpersonal constraints refer to perceived barriers concerning social interactions. In other words, individuals may refuse to travel due to lack of companionship. Yang et al. (2022) state that interpersonal constraints is a critical factor influencing solo travel intentions, but Koronios et al. (2020) revealed that interpersonal constraints may not be very salient in travel decision making. During the current pandemic, many countries and destinations have implemented various control measures, such as social distancing and mandatory quarantine, to prevent a widespread outbreak of the COVID-19 (Li et al., 2020). Given these control measures and personal safety, many people may delay, change, or even terminate their travel plans (Chua et al., 2021). Consequently, it becomes quite difficult to find travel companions. Therefore, some prospective travelers may cancel their travel plans during and after the pandemic. Therefore, the following hypothesis is proposed:

*H2*: Interpersonal constraints negatively influence travel intentions among Malaysian travelers during the COVID-19 pandemic.

Additionally, structural constraints could negatively affect prospective travelers’ intentions to travel as well. Structural constraints are defined as perceived external barriers, including structural cost (i.e., limited financial resources), structural time (i.e., lack of time), and structural space (i.e., place attributes; Nyaupane and Andereck, 2008). These constraints are considered the most influential factors restricting individuals’ participation in leisure activities (Fredman and Heberlein, 2005). Specifically, it is found that prospective travelers’ intentions to travel will decrease under the negative impacts of structural constraints (Lai et al., 2013; Wong and Kuo, 2021). Conversely, Khan et al. (2019b) concluded that structural constraints have no significant relationship with travel intentions as Chen et al. (2021) explained that structural constraints are negotiable. In the current context of the COVID-19 pandemic, structural constraints are arguably more salient and powerful (Hall et al., 2020). Usually, people have to go through health screening or even mandatory quarantine if they need to travel far. As a result, prospective travelers are likely to compromise their travel plans. Therefore, the following hypotheses are proposed:

*H3*: Structural cost negatively influences travel intentions among Malaysian travelers during the COVID-19 pandemic.
*H4*: Structural time negatively influences travel intentions among Malaysian travelers during the COVID-19 pandemic.
*H5*: Structural space negatively influences travel intentions among Malaysian travelers during the COVID-19 pandemic.

Travel constraints do not definitely lead to non-participation but put restrictions on frequency, selection of activity, and destination (Mei and Lantai, 2018). Travel participation is mainly determined by the result of individuals’ negotiation process toward travel (Aziz and Long, 2022; Karl et al., 2022). The tripartite classification of travel constraints is a useful approach to understand the travel negotiation process from intrapersonal, interpersonal, and structural perspectives (Crawford and Godbey, 1987), and it is also important to examine the interplay of the three constraints (Hawkins et al., 1999). Crawford et al. (1991) state that the LCM is organized in a hierarchical and sequential manner, and intrapersonal constraints are the starting point of travel negotiation. Prospective travelers will only consider interpersonal and structural constraints when intrapersonal constraints are not influential (Raymore et al., 1993). However, the sequence may change in different social contexts (Godbey et al., 2010; Hughes et al., 2015). Amid the COVID-19 pandemic, structural constraints become more salient due to various control measures, such as social distancing and mandatory quarantine (Hall et al., 2020; Chua et al., 2021). Meanwhile, it is more difficult to find companions because many people are reluctant to travel with a high level perception of travel constraints. Thus, interpersonal constraints become more conspicuous for some perspective travelers. As a result, prospective travelers are likely to encounter interpersonal and structural constraints before intrapersonal constraints, and their psychological states may be significantly affected by the two categories of constraints even if they are really interested to travel (Li et al., 2020). Besides, individuals’ negotiation process toward travel is largely decided by their own internal psychological factors rather than external ones (Xie and Ritchie, 2019; Karl et al., 2022). Therefore, intrapersonal constraints are the centrality of the LCM, and they probably also mediate the relationship between other constraints and travel intentions (Godbey et al., 2010). Then, the following hypotheses are proposed:

*H6*: Interpersonal constraints negatively influence intrapersonal constraints among Malaysian travelers during the COVID-19 pandemic.
*H7*: Structural cost negatively influences intrapersonal constraints among Malaysian travelers during the COVID-19 pandemic.
*H8*: Structural time negatively influences intrapersonal constraints among Malaysian travelers during the COVID-19 pandemic.
*H9*: Structural space negatively influences intrapersonal constraints among Malaysian travelers during the COVID-19 pandemic.
*H10*: Intrapersonal constraints mediate the negative relationship between interpersonal constraints and travel intentions among Malaysian travelers during the COVID-19 pandemic.
*H11*: Intrapersonal constraints mediate the negative relationship between structural cost and travel intentions among Malaysian travelers during the COVID-19 pandemic.
*H12*: Intrapersonal constraints mediate the negative relationship between structural time and travel intentions among Malaysian travelers during the COVID-19 pandemic.
*H13*: Intrapersonal constraints mediate the negative relationship between structural space and travel intentions among Malaysian travelers during the COVID-19 pandemic.

### Moderating role of gender

Gender goes beyond the limits of biology, and it is also constructed by prevailing social and cultural norms (De Pascale et al., 2022). It is clear from the extant literature that gender has been used for tourism market segmentation, but the relationship between gender and travel behaviors is largely neglected (Huang and van der Veen, 2019). Some past studies suggested that there are significant differences between males and females with regard to perceived travel constraints and travel behaviors due to socially and culturally constructed gender roles (Eagly et al., 2000; Kozak et al., 2007; Khan et al., 2019a). In other words, individuals’ travel related behaviors are often influenced by gendered social and cultural norms (Long et al., 2022a). Significantly, gender, as a socio-psychological construct, could moderate some cognitive mechanisms concerning travel behaviors. Green et al. (2000) found that females perceive more travel risks and face more travel constraints (Nyaupane and Andereck, 2008). Jackson and Henderson (1995) state that females are more likely to be influenced by intrapersonal and interpersonal constraints, whereas males are more likely to encounter structural constraints. Yamashita and Hallmann (2021) revealed that there are obvious gender differences concerning the relationship between travel constraints and behavioral intentions. Specifically, De Pascale et al. (2022) found that the propensity to travel for females with disabilities is more affected by some travel constraints than it is for males with disabilities. It is thought that analyzing gender is crucial to understand and interpret leisure constraints in a selected context (Jackson and Henderson, 1995). During and after the current pandemic, females probably perceive a higher degree of risk in association with travel (Kozak et al., 2007), so how travel constraints influence intentions to travel may vary between prospective male and female travelers. Based on the limited support from the literature, this research intends to further examine the possible moderating role of gender on travel decision making. Therefore, the following hypothesis is proposed:

*H14*: Gender is a moderator in the proposed framework among Malaysian travelers during the COVID-19 pandemic. That is, there are significant differences across gender with regard to H1 to H9 aforementioned.

## Methodology

The authors used a self-administered survey for data collection *via* a convenience sampling method. By following Churchill (1979), the measurement items of this research are adopted or adapted from past studies in tourism literature. The questionnaire is made of three sections. The first section measures travel constraints (structural constraints, 11 items; interpersonal constraints, four items; and intrapersonal constraints, five items), and the measurement items were adapted from Nyaupane and Andereck (2008). The section “Literature review” includes questions for demographic information, such as gender and age. The section “Methodology” contains three items to measure travel intention, which are adapted from Khan et al. (2019a). The rationale to physically separate predictive and criterion variables is to minimize common method bias when the data are collected from a single source (Podsakoff et al., 2012). The survey items are measured by a six-point Likert scale from “1” (strongly disagree) to “6” (strongly agree; Aziz, 2018).

Before collecting data, the authors conducted a small-scale pre-test with 10 Malaysians office workers *via* personal networks to check whether the respondents and the researchers understood the survey questions in the same way. Based on the feedback from the 10 respondents, some items were slightly modified to avoid any possible misunderstandings (Dillman, 2011). Then, the modified survey was sent to another 10 Malaysians office workers for the second round of pre-test, and no further feedback was reported. To expedite the data collection process, this research employed a snowballing sampling technique (Aziz, 2018). The authors persuaded the 20 respondents of the pre-tests who worked in the Klang valley of Malaysia to distribute the modified questionnaire to their colleagues, and 500 questionnaires were given out from June 15 to July 15, 2020.

The rationale to choose office workers as respondents is that they have stable income and thus they are capable to purchase travel-related products. Besides, Klang valley, as the largest metropolitan area of Malaysia, has the highest mean household income in the country (Department of Statistics Malaysia, 2020). Klang valley (i.e., Greater Kuala Lumpur) is located at the center of the west coast of Peninsular Malaysia, and covers the federal territories of Kuala Lumpur, Putrajaya, and nearby cities/towns in the state of Selangor. Meanwhile, this region is diverse with regard to lifestyles and social classes. Thus, some researchers collect data from residents of Klang valley as they believe that these samples are likely to represent the general population of Malaysia (Aziz et al., 2010). Moreover, officer workers with disposable income usually make the purchase decisions and consume travel products/services. Therefore, this research draw samples from office workers of Klang valley.

To check whether the respondents are tourists, the survey has a filter question asking “Have you participated in leisure travel in the last 24 months” in the demographic information section. By the end of July 2020, 360 responses had been received, and three responses were excluded as they did not pass the filter question. Among the 357 usable responses, 163 and 194 were from males and females, respectively. This research targeted office workers for data collection because they are likely to purchase travel products with stable income.

## Data analysis

The descriptive analysis of this study is summarized in Table 1. According to the mean values of the measurement items, it is found that males perceive slightly higher structural cost and time than females, and females perceive slightly higher structural space than males. The respondents generally perceive low interpersonal and intrapersonal constraints regardless of gender. More importantly, females have slightly higher intentions to travel than their male counterparts, which is consistent with the fact that males perceive higher intrapersonal constraints than females.

**Table 1:** Descriptive analysis.

Construct/Associated items	Male		Female		Total	
	Mean value	Standard deviation	Mean value	Standard deviation	Mean value	Standard deviation
**Structural Cost (SC)**	**3.581**		**3.429**		**3.499**	
I do not have enough money to travel.	3.540	1.602	3.397	1.419	3.462	1.507
The things I want to do are expensive.	3.767	1.468	3.758	1.395	3.762	1.429
I could not afford to travel.	3.515	1.492	3.098	1.338	3.289	1.425
Travel is too expensive.	3.503	1.463	3.464	1.465	3.482	1.464
**Structural Time (ST)**	**3.662**		**3.624**		**3.641**	
I have no time to travel.	3.644	1.530	3.531	1.503	3.583	1.516
Family commitments keeps me from traveling.	3.515	1.595	3.412	1.664	3.459	1.634
My current work schedule is too tight to travel.	3.828	1.573	3.928	1.528	3.882	1.549
**Structural Space (SS)**	**3.681**		**3.719**		**3.702**	
The destination that I want to go is far from my home.	4.018	1.557	4.237	1.498	4.137	1.529
There are no areas nearby I want to visit.	3.417	1.522	3.139	1.545	3.266	1.541
The places nearby where I want to go is crowded with people.	4.043	1.411	4.232	1.333	4.146	1.372
The weather of the place where I want to visit is not favorable.	3.245	1.379	3.268	1.485	3.258	1.438
**Interpersonal Constraints (IPTC)**	**2.976**		**2.978**		**2.977**	
I have no companions to travel with.	2.393	1.533	2.479	1.590	2.440	1.565
My friends/family prefer to travel to other places.	3.067	1.406	3.062	1.442	3.064	1.426
It is not fun to travel by myself.	3.730	1.883	3.814	1.835	3.776	1.858
My friends/family are not interested in traveling.	2.712	1.501	2.557	1.392	2.627	1.445
**Intrapersonal Constraints (ITTC)**	**3.118**		**2.644**		**2.861**	
I do not have a great deal of interest to travel.	3.000	1.498	2.381	1.369	2.664	1.463
I am not knowledgeable about where to travel.	2.853	1.466	2.459	1.332	2.639	1.409
Travel involves too much risk.	3.785	1.661	3.660	1.617	3.717	1.638
I am not interested in traveling.	2.773	1.633	2.113	1.338	2.415	1.516
Travel is not a major interest to me.	3.178	1.676	2.608	1.523	2.868	1.620
**Travel Intentions (IN)**	**4.198**		**4.278**		**4.242**	
I am likely to travel locally or internationally within next 2 years.	4.098	1.512	4.088	1.421	4.092	1.463
I intent to travel locally or internationally within next 2 years.	3.933	1.441	4.134	1.367	4.042	1.405
I want to travel locally or internationally within next 2 years.	4.564	1.366	4.613	1.335	4.591	1.350

This research employed the partial least squares structural equation modeling (PLS-SEM), and software SmartPLS 3.3.3 was adopted for data analysis. PLS-SEM, as a multivariate analysis approach, is suitable to perform multigroup analysis (MGA) to examine whether there are significant differences across gender in relation to the proposed structural paths (Henseler et al., 2016). In addition, it is found that the collected data are abnormally distributed by using an online tool available at https://webpower.psychstat.org/wiki/tools/index, which further justifies the application of PLS-SEM (Hair et al., 2017). With regard to the minimum sample size of PLS-SEM, Reinartz et al. (2009) argues that 100 is the threshold. G*Power was also used to calculate the minimum sample size for the proposed framework. The sample size of this study (i.e., male and female groups) reaches the minimum threshold for data analysis *via* PLS-SEM (Hair et al., 2017).

### Measurement model

By following Hair et al. (2017), this research examined outer loadings, composite reliability (CR), average extracted (AVE), and heterotrait-monotrait (HTMT) to confirm the reflective measurement model (complete and split datasets of male and female). As shown in Table 2, the CR values ranged between 0.70 and 0.95, which indicates internal consistency of the items (Chin, 2010). To confirm the convergent validity of the research, an item’s outer loading has to be no less than 0.40 and its associated AVE has to be higher than 0.50 (Hulland, 1999), so the third item measuring interpersonal travel constraints was deleted.

**Table 2:** Internal consistency and convergent validity.

Construct/Item	Loading			CR			AVE		
	Male	Female	Complete	Male	Female	Complete	Male	Female	Complete
**SC**				0.937	0.910	0.923	0.789	0.718	0.749
SC1	0.905	0.877	0.891						
SC2	0.861	0.767	0.808						
SC3	0.907	0.909	0.909						
SC4	0.878	0.83	0.851						
**ST**				0.874	0.86	0.868	0.701	0.671	0.687
ST1	0.863	0.834	0.845						
ST2	0.927	0.806	0.875						
ST3	0.706	0.818	0.762						
**SS**				0.812	0.812	0.807	0.526	0.533	0.523
SS1	0.592	0.544	0.546						
SS2	0.803	0.908	0.872						
SS3	0.624	0.547	0.561						
SS4	0.848	0.845	0.849						
**IPTC**				0.802	0.828	0.817	0.577	0.618	0.599
IPTC1	0.736	0.811	0.776						
IPTC2	0.684	0.715	0.705						
IPTC4	0.849	0.827	0.835						
**ITTC**				0.871	0.879	0.878	0.58	0.604	0.597
ITTC1	0.810	0.859	0.841						
ITTC2	0.739	0.787	0.776						
ITTC3	0.565	0.432	0.494						
ITTC4	0.822	0.884	0.854						
ITTC5	0.838	0.835	0.839						
**IN**				0.819	0.872	0.839	0.614	0.699	0.649
IN1	0.912	0.900	0.920						
IN2	0.869	0.927	0.918						
IN3	0.505	0.654	0.507						

CR, Composite Reliability; AVE, Average Variance Explained.

Besides, the Heterotrait and Monotrait (HTMT) ratio of correction method is adopted to assess discriminant validity on complete and split datasets of male and female (Henseler et al., 2016). As shown in Table 3, all the HTMT values are lower than the threshold of 0.90, which indicates there is no multi-collinearity issue between latent variables of this research (Henseler et al., 2016).

**Table 3:** Discriminant validity.

Data Set	Construct	IN	IPTC	ITTC	SC	SS
Male	IPTC	0.173				
	ITTC	0.312	0.528			
	SC	0.118	0.256	0.317		
	SS	0.145	0.283	0.397	0.308	
	ST	0.066	0.173	0.216	0.168	0.306
Female	IPTC	0.108				
	ITTC	0.171	0.727			
	SC	0.137	0.564	0.611		
	SS	0.183	0.353	0.349	0.236	
	ST	0.096	0.302	0.38	0.241	0.261
Complete	IPTC	0.075				
	ITTC	0.224	0.621			
	SC	0.103	0.416	0.462		
	SS	0.157	0.305	0.361	0.261	
	ST	0.053	0.226	0.291	0.204	0.282

Discriminant validity established at HTMT _0.90_.

With regard to the assessment of Goodness-of-Fit (GoF), Standardized Root Mean Square Residual (SRMR) was used as a measure. As shown in Table 4, the SRMR value of male, female, and complete datasets are 0.077, 0.080, and 0.068, respectively. All the values are no more than the threshold at 0.080, so all the three datasets satisfy the requirements for GoF (Henseler and Sarstedt, 2013).

**Table 4:** Model Fit using SRMR.

Data Set	SRMR
Male	0.077
Female	0.080
Complete	0.068

Then, lateral collinearity (i.e., predictor-criterion collinearity) issue have to be ruled out before assessing the structural model of the research. Although the discriminant validity (vertical collinearity) of the research has been confirmed, lateral collinearity issue may exist and sometimes distort the findings (Kock and Lynn, 2012). Thus, Variance Inflation Factor (VIF) is assessed identify possible multi-collinearity issues. As shown in Table 5, all the VIF scores are no higher than the threshold of 3.3, indicating the current study is not likely to have collinearity issue (Diamantopoulos and Siguaw, 2006).

**Table 5:** Collinearity test (VIF).

Construct	IN			ITTC		
	Male	Female	Complete	Male	Female	Complete
IPTC	1.238	1.598	1.376	1.079	1.329	1.179
ITTC	1.403	1.830	1.539			
SC	1.123	1.455	1.248	1.096	1.272	1.160
SS	1.224	1.155	1.176	1.134	1.125	1.128
ST	1.089	1.117	1.102	1.083	1.103	1.094

VIF, Variance Inflation Factor.

### Structural model

To confirm the structural model of the current research, the authors conducted a bootstrapping procedure with 5,000 resamples to estimate the significance of the path coefficient (Hair et al., 2017). The path coefficients for the complete dataset are shown in Table 6. Based on the results, H1, H6, H7, H9, H10, H11, and H13 are supported at 95% CIs with one-tailed testing, and the rest are not supported. Specifically, only intrapersonal constraints directly influence travel intentions, interpersonal constraints, and structural constraints (except structural time) indirectly influence travel intentions *via* intrapersonal constraints. Meanwhile, interpersonal and structural constraints (except structural time) impose direct effects on intrapersonal constraints. Concerning the significant relationships (H1, H6, H7, and H9), it is necessary to check their effect sizes (*f* ^2^; Hair et al., 2017). According to the *f* ^2^ values (see Table 7), it is found that interpersonal constraints (*f* ^2^ = 0.167) has a medium effect size on intrapersonal constraints; structural cost (*f* ^2^ = 0.076) and structural space (*f* ^2^ = 0.042) have a small effect size on intrapersonal constraints; intrapersonal constraints (*f* ^2^ = 0.038) have a small effect size on travel intentions for the complete dataset. Table 6 also shows information on the coefficient of determination (*R* ^2^) and predictive relevance (*Q* ^2^) of the exogenous variables and endogenous variables. The *R* ^2^ values of intrapersonal constraints and travel intentions are 0.350 and 0.048, respectively, for the complete dataset, indicating the model has a moderate and weak level of predictive accuracy (Hair et al., 2017). Lastly, all the *Q*^2^ values are greater than 0, which indicates acceptable predictive quality of the model (Hair et al., 2017).

**Table 6:** Results of hypotheses testing.

Hypothesis	Relationship	Beta	Std error	T Value	*p* Value	LL	UL	Supported
H1	ITTC - > IN	−0.236	0.084	2.811	0.002	−0.354	−0.077	Yes
H2	IPTC - > IN	0.079	0.068	1.163	0.122	−0.030	0.194	No
H3	SC - > IN	0.010	0.072	0.139	0.445	−0.112	0.123	No
H4	ST - > IN	0.050	0.060	0.830	0.203	−0.059	0.140	No
H5	SS - > IN	−0.052	0.081	0.639	0.261	−0.178	0.092	No
H6	IPTC - > ITTC	0.358	0.053	6.805	0.000	0.268	0.440	Yes
H7	SC - > ITTC	0.240	0.052	4.584	0.000	0.154	0.325	Yes
H8	ST - > ITTC	0.074	0.044	1.684	0.046	−0.006	0.139	No
H9	SS - > ITTC	0.175	0.048	3.657	0.000	0.093	0.253	Yes
H10	IPTC - > ITTC - > IN	−0.084	0.032	2.601	0.005	−0.136	−0.033	Yes
H11	SC - > ITTC - > IN	−0.057	0.024	2.327	0.010	−0.100	−0.022	Yes
H12	ST - > ITTC - > IN	−0.017	0.014	1.271	0.102	−0.045	−0.001	No
H13	SS - > ITTC - > IN	−0.041	0.020	2.077	0.019	−0.077	−0.013	Yes

LL (lower limit) and UL (upper limit) at 95 percent confidence intervals.

**Table 7:** *R*^2^, *f*^2^, and *Q*^2^.

Data Set	Construct	*R^2^*	*f ^2^*		*Q^2^*
			IN	ITTC	
Male	IPTC		0.016	0.147	
	ITTC	0.287	0.083		0.144
	SC		0.002	0.025	
	SS		0.001	0.079	
	ST		0.003	0.005	
	IN	0.087			0.033
Female	IPTC		0.000	0.202	
	ITTC	0.454	0.014		0.252
	SC		0.006	0.144	
	SS		0.011	0.026	
	ST		0.004	0.012	
	IN	0.035			0.005
Complete	IPTC		0.005	0.167	
	ITTC	0.350	0.038		0.194
	SC		0.000	0.076	
	SS		0.002	0.042	
	ST		0.002	0.008	
	IN	0.048			0.016

### Multi-group analysis

Before comparing the proposed structural paths across gender, the measurement invariance of composites (MICOM) has to be conducted to confirm the measurement invariance of the reserach (Rasoolimanesh et al., 2017). Henseler et al. (2016) explained that there are three steps involved for MICOM, including (1) the assessment of configural invariance, (2) assessment of compositional invariance, and (3) assessment of equal composite means and variances. As shown in Table 8, the first and the second steps of MICOM procedure are confirmed, and the third step is not supported. Therefore, a partial measurement invariance between male and female groups is established, and which is adequate to compare the path coefficients across gender by MGA (Henseler et al., 2016).

**Table 8:** Results of invariance measurement testing using permutation.

Construct	Configural invariance (Same algorithms for both groups)	Compositional invariance (Correlation = 1)		Equal mean value		Equal variance	
		C = 1	Confidence Interval (CIs)	Differences	Confidence Interval (CIs)	Differences	Confidence Interval (CIs)
IN	Yes	0.989	[0.830, 1.000]	0.075	[−0.175, 0.173]	0.000	[−0.237, 0.244]
IPTC	Yes	0.996	[0.977, 1.000]	−0.037	[−0.172, 0.179]	0.022	[−0.216, 0.224]
ITTC	Yes	0.999	[0.993, 1.000]	−0.431	[−0.174, 0.180]	−0.176	[−0.229, 0.235]
SC	Yes	1.000	[0.995, 1.000]	−0.141	[−0.176, 0.179]	−0.234	[−0.196, 0.200]
SS	Yes	0.992	[0.917, 1.000]	−0.037	[−0.178, 0.178]	0.119	[−0.209, 0.220]
ST	Yes	0.978	[0.919, 1.000]	−0.046	[−0.175, 0.172]	−0.078	[−0.194, 0.202]

Then, a bootstrapping procedure with 5,000 re-samples was conducted to compare differences between the two groups (Hair et al., 2017). Based on Table 9, some interesting findings are revealed. Firstly, structural space (SS) and interpersonal travel constraints (IPTC) have a positive effect on intrapersonal travel constraints (ITTC) in both data groups. Secondly, structural cost (SC) has a positive effect on ITTC only among females, and ITTC has a negative effect on travel intentions (IN) only among males. Thirdly, ST has no significant influence on ITTC, and SC, ST, SS, and IPTC have no significant influence on IN regardless of gender. These results are also verified by coefficient of determination (*R*^2^), effect size (*f* ^2^), and predictive relevance (*Q*^2^) indicated in Table 6 (Hair et al., 2017). Therefore, there are significant differences between the effects of SC on ITTC (H1) and between the effects of ITTC on IN (H9) across gender. Based on the findings, H14 is partially supported.

**Table 9:** Results of hypothesis testing.

Hypothesis	Beta Male	Beta Female	CIs Male	CIs Female	Beta Differences	Value of *p* (MGA)	Supported
SC - > ITTC	0.140^**^	0.316^***^	[−0.003, 0.269]	[0.221, 0.413]	0.176	0.040^**^	Yes
ST - > ITTC	0.064	0.087^**^	[−0.154, 0.158]	[−0.003, 0.164]	0.023	0.415	No
SS - > ITTC	0.254^***^	0.128^**^	[0.107, 0.363]	[0.022, 0.216]	0.126	0.904	No
IPTC - > ITTC	0.337^***^	0.383^***^	[0.197, 0.460]	[0.271, 0.480]	0.046	0.326	No
SC - > IN	−0.041	0.093	[−0.189, 0.138]	[−0.149, 0.251]	0.134	0.196	No
ST - > IN	0.058	0.069	[−0.136, 0.196]	[−0.115, 0.218]	0.011	0.471	No
SS - > IN	0.029	−0.111	[−0.146, 0.205]	[−0.264, 0.134]	0.140	0.814	No
IPTC - > IN	0.134	−0.001	[−0.046, 0.287]	[−0.179, 0.167]	0.134	0.823	No
ITTC - > IN	−0.325^***^	−0.156	[−0.468, −0.119]	[−0.353, 0.109]	0.170	0.048^**^	Yes

** *p* < 0.05 and

*** *p* < 0.01.

## Discussion and conclusion

The current COVID-19 pandemic has imposed numerous challenges to the tourism industry, and travel constraints become very salient for prospective travelers. Through a critical interrogation on the literature of travel constraints, this study aims to gain a better understanding of the relationship between travel constraints and travel decision making by revisiting the Leisure Constraints Model (LCM) amid a global health crisis. Meanwhile, the role of gender is considered with regard to the interplay between travel constraints and travel intentions. This research adopted a self-administered survey, and collected data from Malaysians office workers in Klang valley. PLS-SEM was used for conducting path analysis and multi-group analysis (MGA) for hypothesis testing. Based on the results of statistical analysis, the revised Leisure Constraints Model (LCM) is valid to explain and predict Malaysian tourists’ travel behavior. The empirical findings of the research provide theoretical and practical implications to tourism marketing literature, which are meaningful for related stakeholders for tourism recovery.

### Theoretical implications

By revisiting the Leisure Constraints Model (LCM), it is found that structural constraints (except structural time) and interpersonal constraints have a significant impact on intrapersonal constraints; intrapersonal constraints significantly influence travel intentions; intrapersonal constraints mediate the relationship between structural constraints (except structural space), interpersonal constraints, and travel intentions among Malaysian tourists. Besides, the empirical findings indicate that there are salient differences across gender with regard to the relationship between structural cost and intrapersonal constraints, and the relationship between intrapersonal constraints and travel intentions. Based on the aforementioned findings, this research makes some theoretical contributions to the body of tourism knowledge, especially concerning travel decision making in the current COVID-19 pandemic.

Firstly, it is confirmed that the sequence of the Leisure Constraints Model (LCM) varies in different social contexts (Hughes et al., 2015), which contradicts some early research, such as Crawford et al. (1991) and Raymore et al. (1993). As per the original Leisure Constraints Model (LCM), individuals’ cognitive negotiation process on whether to participate in travel activities begin from intrapersonal constraints, then the negation process moves to structural and interpersonal constraints only when intrapersonal constraints are basically solved (Crawford et al., 1991). Specifically, prospective travelers have to get over intrapersonal constraints (e.g., attitude) before they can think over factors related to structural constraints (e.g., cost) and interpersonal constraints (e.g., social interactions). However, the starting point of the travel negotiation process is fluid rather than static in various social settings (Godbey et al., 2010). In the context of the COVID-19 pandemic, the sequence of the Leisure Constraints Model (LCM) changed from intrapersonal constraints to interpersonal and structural constraints. Due to related control measures, such as social distancing and mandatory quarantine, interpersonal and structural constraints become even more influential, especially in international travel (Hall et al., 2020). Consequently, individuals have to overcome interpersonal and structural constraints before intrapersonal constraints. Alternatively, prospective travelers’ psychological states are very likely to be influenced by external barriers and social interactions with other people even if they desire to travel.

Secondly, it is reaffirmed that intrapersonal constraints play a significantly central role in the Leisure Constraints Model (LCM; Xie and Ritchie, 2019). Structural and interpersonal constraints do not have direct impacts on travel intentions though their impacts are literally magnified by the current pandemic. On the contrary, they (except structural time) impose indirect effects *via* intrapersonal constraints. That is, intrapersonal constraints mediate the negative relationship between these constraints and travel intentions, which suggests that structural and interpersonal constraints are not the determining factors influencing prospective travelers’ travel behaviors even if they are the starting point of the travel negotiation process. In other words, prospective travelers are unlikely to abandon their travel plans simply due to external barriers and lack of companionship to travel. Nevertheless, these constraints impose their impacts on travel intentions indirectly *via* prospective travelers’ inner psychological states (Chua et al., 2021). Out of expectation, structural time does not negatively affect travel intentions, and it does not influence travel intentions indirectly *via* intrapersonal constraints either. One possible explanation is that the data was collected in 2020 when Malaysians were not allowed to travel abroad for leisure purposes and most overseas destinations required mandatory quarantine (Hall et al., 2020). In such a case, barriers related to cost and space are more salient than structural time for many prospective travelers.

Thirdly, this research verifies that gender plays a significant role with regard to perception of travel constraints and travel decision making. By conducting multigroup analysis (MGA), it is found that gender moderates the positive association between structural cost and intrapersonal constraints, and the negative association between intrapersonal constraints and travel intentions. Comparatively, females perceived cost as a main structural constraints influencing their inner psychological attributes toward travel (i.e., intrapersonal constraints), and males perceived intrapersonal constraints as a dominant factor determining their travel intentions. Interestingly, intrapersonal constraints do not negatively influence travel intentions among females, which may be attributed to the fact that some consumers are likely to make purchase decisions mainly based on their attitudinal evaluations (Long et al., 2022b). Specifically, Uatay et al. (2019) found that intrapersonal constraints affect travel intentions *via* attitude for female travelers. There are no significant differences across gender concerning other proposed relationship of the research. These findings contradict some previous studies, such as Jackson and Henderson (1995) who argue that males are very likely to be influenced by structural constraints and females are more concerned about interpersonal and intrapersonal constraints.

Meanwhile, this study confirms that it is important to comprehend travel decision making from a gender perspective (Yamashita and Hallmann, 2021) as human behaviors, to some extent, are framed by socially and culturally constructed gender roles (Kozak et al., 2007; Khan et al., 2019a; Long et al., 2022a). Therefore, prospective travelers may have different perceptions about travel constraints and the effects of travel constraints on travel intentions, which highlight the necessity to examine the effect of gender in travel decision making. Based on limited literature supports, the current research attests that gender moderates the process of tourism decision making with empirical evidence. Therefore, the study makes a notable contribution in attracting attention to gender gap in the tourism literature. Moreover, we appeal for further investigations into gender differences in travel behaviors.

### Practical implications

As per the theoretical contributions aforementioned, this research offers a few practical implications to tourism policy makers, destination marketers and related businesses. The empirical findings suggest that the starting point of the travel negotiation process shifted from intrapersonal constraints to structural and interpersonal constraints in the context of the COVID-19 pandemic; the latter two types of constraints impose indirect effects on travel intentions (mediated by intrapersonal constraints); and gender role influences how individuals perceive travel constraints. Therefore, it is significant to revisit the interplay of travel constraints, gender role and travel intentions amid the “new normal.”

Firstly, tourism policy makers and related stockholders are advised to collaborate for reducing external barriers to travel (i.e., cost and time). As a matter of fact, some destinations, such as Thailand and Singapore, have already removed PCR test requirements and eased mandatory quarantine for vaccinated travelers (Mandal, 2022). To minimize the negative impacts of structural constraints and boost quick tourism recovery, destinations may take further efforts to exempt vaccinated travelers with valid medical insurance from mandatory quarantine if they are from countries/regions with low COVID-19 cases. With a perception of low external barriers, individuals are more likely to travel again, especially travel abroad.

Secondly, tourism authorities, destination marketers, and related businesses should take effective measures to ensure travelers’ health and make them believe that it is safe to travel to the destination. The COVID-19 pandemic has arguably changed many people’s travel preferences and related behaviors (Wen et al., 2020). Prospective travelers may have more health concerns when they ponder whether they should travel and where to travel. Thus, solely reducing structural constraints (i.e., cost and time) is far from enough to restore individuals’ confidence in travel. Even if some prospective travelers do not have a chance to travel in the near future, their opinions, as a form of interpersonal constraints, may influence their family and friends’ travel behaviors. Without sufficient support or companionship, prospective travelers may feel anxious about travel and eventually give their travel plans (Chua et al., 2021).

Thirdly, destination marketers and tourism businesses should be aware of gendered differences with regard to perception of travel constraints and their effects on travel behaviors. Females are more concerned about structural cost and its effect on intrapersonal constraints is significant; and males are more concerned about intrapersonal constraints and its effect on travel intentions is significant. Based on these findings, destination marketers and tourism businesses may adjust their marketing strategies and travel products accordingly.

### Limitation and future research

This research provides new insights on travel decision making amid the COVID-19 pandemic by revisiting the Leisure Constraints Model (LCM), but it has some limitations. Firstly, this study collected data from office workers of Klang valley by convenience sampling method, so it should be cautious to generalize the findings concerning tourists in other parts of Malaysia and the rest of the world. Future research is advised to collect data from different states/regions of Malaysia for improving data representativeness, and also examine whether there are significant differences among Malaysian tourists from different states/regions. Besides, future research is suggested to collect longitudinal data to compare if there are significant differences with regard to Malaysian tourists’ perception toward travel constraints and intentions to travel during and after the current global health crisis. Secondly, this study only examine the effects of travel constraints and gender role on travel intentions, but some other factors also have important effects on travel decision making, such as perceived risk (Aziz and Long, 2022), destination image (Caber et al., 2020), and crisis management (Rastegar et al., 2021). Thirdly, this study examines behavioral intentions rather than actual behaviors. It should be noted that people may not always behave in line with corresponding behavioral intentions (Ajzen, 2020). Thus, future research should expand the current research framework to actual travel behaviors.

## Data availability statement

The raw data supporting the conclusions of this article will be made available by the authors, without undue reservation.

## Ethics statement

Ethical review and approval was not required for the study on human participants in accordance with the local legislation and institutional requirements. The patients/participants provided their written informed consent to participate in this study.

## Author contributions

NA: supervision, data collection, and funding acquisition. FL: research idea, conceptualization, and writing–original draft preparation. MB and MR: revision and editing. All authors contributed to the article and approved the submitted version.

## Funding

This research is supported by UKM-GSB (project serial number: GSB-2021-009).

## Conflict of interest

The authors declare that the research was conducted in the absence of any commercial or financial relationships that could be construed as a potential conflict of interest.

## Publisher’s note

All claims expressed in this article are solely those of the authors and do not necessarily represent those of their affiliated organizations, or those of the publisher, the editors and the reviewers. Any product that may be evaluated in this article, or claim that may be made by its manufacturer, is not guaranteed or endorsed by the publisher.
